# Activation or exhaustion of CD8^+^ T cells in patients with COVID-19

**DOI:** 10.1038/s41423-021-00750-4

**Published:** 2021-08-19

**Authors:** Min-Seok Rha, Eui-Cheol Shin

**Affiliations:** 1 Laboratory of Immunology and Infectious Diseases, Graduate School of Medical Science and Engineering, Korea Advanced Institute of Science and Technology (KAIST), Daejeon, Republic of Korea; 2 Department of Otorhinolaryngology, Yonsei University College of Medicine, Seoul, Republic of Korea; 3 The Center for Epidemic Preparedness, KAIST, Daejeon, Republic of Korea

**Keywords:** CD8^+^ T cell, Activation, T-cell exhaustion, SARS-CoV-2, COVID-19, Cellular immunity, Infection

## Abstract

In addition to CD4^+^ T cells and neutralizing antibodies, CD8^+^ T cells contribute to protective immune responses against SARS-CoV-2 in patients with coronavirus disease 2019 (COVID-19), an ongoing pandemic disease. In patients with COVID-19, CD8^+^ T cells exhibiting activated phenotypes are commonly observed, although the absolute number of CD8^+^ T cells is decreased. In addition, several studies have reported an upregulation of inhibitory immune checkpoint receptors, such as PD-1, and the expression of exhaustion-associated gene signatures in CD8^+^ T cells from patients with COVID-19. However, whether CD8^+^ T cells are truly exhausted during COVID-19 has been a controversial issue. In the present review, we summarize the current understanding of CD8^+^ T-cell exhaustion and describe the available knowledge on the phenotypes and functions of CD8^+^ T cells in the context of activation and exhaustion. We also summarize recent reports regarding phenotypical and functional analyses of SARS-CoV-2-specific CD8^+^ T cells and discuss long-term SARS-CoV-2-specific CD8^+^ T-cell memory.

## Introduction

Since the initial reports of pneumonia cases of unknown origin in Wuhan, China, in late December 2019 [1], novel severe acute respiratory syndrome coronavirus 2 (SARS-CoV-2) has been rapidly spreading worldwide. Coronavirus disease 2019 (COVID-19), caused by SARS-CoV-2 infection, manifests with a broad spectrum of clinical symptoms, from asymptomatic infection to critical disease [2]. COVID-19 has threatened public health and had a devastating economic impact. Global efforts are underway to control the COVID-19 pandemic. Prophylactic COVID-19 vaccines using various platforms have been approved since December 2020, and their administration has started in populations throughout the world [3–7].

A better understanding of host immune responses to SARS-CoV-2 is crucial to the development of effective vaccines and therapeutics and ending the current pandemic. SARS-CoV-2 infection elicits the activation of both innate and adaptive immunity [8–11]. In adaptive immunity, CD8^+^ T cells play an essential role in controlling viral infection by killing virus-infected cells and producing effector cytokines. Since the emergence of COVID-19, remarkable progress has been made in understanding CD8^+^ T-cell responses against SARS-CoV-2. It is now clear that SARS-CoV-2-specific CD8^+^ T-cell responses are detected in the acute and convalescent phases of COVID-19 [12–17]. In addition, recent studies using animal models have reported that CD8^+^ T cells contribute to protection from the development of severe COVID-19 [18, 19].

In COVID-19 patients, the CD8^+^ T-cell population undergoes quantitative and qualitative changes. Decreased cell number and activation phenotypes are frequently observed, particularly in severe disease [16, 20–24]. Previous studies have also reported exhaustion phenotypes of CD8^+^ T cells in patients with severe COVID-19 based on the upregulation of inhibitory receptors (IRs) [20, 25–30], which may impair host defenses and result in poor disease outcomes. In contrast, no significant evidence of CD8^+^ T-cell exhaustion has been observed in several single-cell RNA sequencing (scRNA-seq) analyses [31, 32]. However, all of these studies have the limitation of their conclusions relying on the expression of IRs or transcripts related to T-cell exhaustion without information on the antigen specificity of CD8^+^ T cells and their effector functions. Our previous study using major histocompatibility complex class I (MHC-I) multimers demonstrated that PD-1^+^ SARS-CoV-2-specific CD8^+^ T cells are functionally active in terms of interferon (IFN)-γ production, implying that these cells are not truly exhausted [33].

Several reviews have already summarized and discussed different aspects of CD8^+^ T-cell responses to SARS-CoV-2 in terms of cross-reactivity, kinetics, and protective roles [34–39]. In the current review, we focus on the activation and exhaustion of CD8^+^ T cells in patients with COVID-19. We summarize the current understanding of CD8^+^ T-cell exhaustion and discuss available knowledge regarding the activation and exhaustion of CD8^+^ T cells in the context of COVID-19.

## The characteristics of exhausted CD8^+^ T cells

### An overview of CD8^+^ T-cell exhaustion

During acute viral infection, naive CD8^+^ T cells that recognize antigens presented on MHC-I by their T-cell receptors (TCRs) are activated and undergo clonal expansion and differentiation into effector CD8^+^ T cells [40, 41]. Effector CD8^+^ T cells produce cytokines, including IFN-γ and tumor necrosis factor (TNF), and directly kill target cells [42]. In the subsequent contraction phase following antigen clearance, a small proportion of effector CD8^+^ T cells differentiate into memory CD8^+^ T cells [40, 41]. Memory CD8^+^ T cells rapidly exert effector functions upon antigen re-encounter, playing a crucial role in host protection during reinfection [41].

**Fig. 1:**
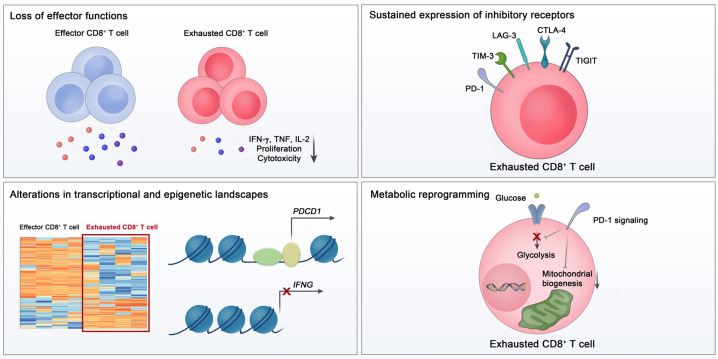
Key features of exhausted CD8^+^ T cells. Exhausted CD8^+^ T cells are characterized by a loss of effector functions, sustained expression of inhibitory receptors, altered transcriptional and epigenetic landscape, and metabolic reprogramming

On the other hand, when antigens persist in chronic viral infection or cancer, the development of memory CD8^+^ T cells fails, and the effector functions of CD8^+^ T cells become impaired [43, 44]. This state of CD8^+^ T cells is called “exhaustion.” CD8^+^ T-cell exhaustion was first reported in a previous study using a mouse model of chronic lymphocytic choriomeningitis virus (LCMV) infection [45]. LCMV-specific CD8^+^ T cells that are continuously stimulated by antigens exhibit impaired effector functions and limited proliferation compared to conventional memory CD8^+^ T cells [46]. These findings have also been observed in human patients with chronic viral infection or cancer [47, 48]. T-cell exhaustion is evidently the main mechanism underlying immune dysfunction during chronic viral infection and cancer [43, 44, 49], and virus antigen-specific and tumor antigen-specific CD8^+^ T cells exhibit features of T-cell exhaustion and dysfunction [47, 48, 50–53]. CD8^+^ T cell exhaustion is now considered a distinct differentiation state of CD8^+^ T cells, with several key features (Fig. 1).

### The loss of effector function

CD8^+^ T-cell exhaustion is characterized by progressive and hierarchical impairment of effector functions. Generally, IL-2 production and proliferative capacity become compromised early, followed by defects in TNF production and cytotoxicity [54]. The loss of IFN-γ production occurs in more severely exhausted CD8^+^ T cells [55]. When antigen stimulation is excessive, clonal deletion or apoptosis of antigen-specific CD8^+^ T cells occurs, which is considered the end stage of CD8^+^ T-cell exhaustion [54]. Functional loss of exhausted CD8^+^ T cells eventually results in a failure to eliminate the virus or tumor cells. Therefore, a correlation between viral load and the severity of exhaustion in chronic viral infection can be explained by functional impairment of exhausted CD8^+^ T cells. Furthermore, exhausted CD8^+^ T cells respond poorly to homeostatic cytokines, including IL-7 and IL-15 [56], in relation to their low expression of CD127 and CD122 [57].

The functions of exhausted CD8^+^ T cells may vary across diseases, possibly related to antigens and the immune microenvironment. The absence of CD4^+^ cells has been shown to contribute to CD8^+^ T-cell exhaustion [58, 59]. In addition, a recent study reported that hypoxia, which is frequently observed in cancer, promotes functional impairment of T cells in the presence of continuous TCR stimulation [60].

### Sustained expression of inhibitory receptors

Another key feature of exhausted CD8^+^ T cells is sustained expression of IRs [43, 44]. IRs counteract T-cell activation to avoid exaggerated immune activation. In particular, in antigen-persisting conditions, IRs mediate T-cell exhaustion by negatively regulating the activation of antigen-specific T cells.

Among the various IRs, PD-1 is a key molecule responsible for T-cell exhaustion [43, 44]. PD-1 is a transmembrane glycoprotein receptor belonging to the CD28 family [61]. An immunoreceptor tyrosine-based inhibitory motif and an immunoreceptor tyrosine-based switch motif are located in the intracellular region of PD-1 [44]. PD-1 has two ligands: PD-L1 (CD274 or B7-H1) and PD-L2 (B7-DC) [62]. PD-L1 is expressed not only by immune cells but also by nonimmune cells, including tumor cells, whereas PD-L2 is mainly expressed by antigen-presenting cells [63]. In the case of T cells, PD-1 expression is mainly induced and sustained by TCR-mediated stimulation, but PD-1 expression can also be induced by cytokines and other stimuli [62]. PD-1/PD-L1 engagement inhibits T-cell activation via the recruitment of SHP-2 and subsequent dephosphorylation of signaling molecules [43, 64, 65]. PD-1 blockade has been demonstrated to reinvigorate exhausted CD8^+^ T cells and reduce viral load during chronic LCMV infection [66, 67]. In tumor models, the blockade of PD-1 signaling also enhances the functions of CD8^+^ T cells, with robust antitumor effects [68, 69]. On the basis of these results, cancer immunotherapy targeting PD-1 has been developed and shown to have clinical benefits in multiple types of cancer [70–74].

In addition to PD-1, exhausted T cells express a battery of IRs, including TIM-3, LAG-3, TIGIT, and CTLA-4 [75, 76]. Although individual expression of PD-1 or other IRs is not sufficient to indicate CD8^+^ T-cell exhaustion, the coexpression of multiple IRs is considered a main characteristic of exhaustion. In exhausted CD8^+^ T cells, several IRs are coexpressed with PD-1 and provide a synergistic inhibitory effect [53, 77, 78]. Exhausted CD8^+^ T cells with a higher number of coexpressed IRs have more severe exhaustion [77]. Simultaneous blockade of multiple IRs leads to robust reinvigoration of exhausted T cells in cancers and chronic viral infections [70, 76].

### Changes in the epigenetic and transcriptional landscape

In exhausted virus-specific CD8^+^ T cells from chronically LCMV-infected mice, the expression of multiple genes is altered, including genes related to TCR and cytokine signaling pathways, costimulatory pathways, and energy metabolism, as well as genes encoding IRs and transcription factors [79]. Several studies using a mouse model of chronic LCMV infection have also shown that various transcription factors, including T-bet, Eomes, Blimp1, NFAT, TCF1, IRF4, and TOX, are involved in CD8^+^ T-cell exhaustion [80–86]. In addition, BATF, which is commonly upregulated in both HIV-specific CD8^+^ T cells from HIV progressors and Jurkat cells following PD-1 ligation, mediates PD-1-induced suppression of T cells in vivo [87]. Although a master transcription factor specific to exhaustion has not yet been identified, multiple transcription factors are associated with exhaustion-specific gene expression and function [80, 81, 87, 88].

Epigenetic regulation at the chromatin level also plays an important role in controlling the differentiation and fate of CD8^+^ T cells. Recent technological advances in epigenetics have enabled us to investigate the epigenetic characteristics of exhausted CD8^+^ T cells. Previous studies using an assay for transposase-accessible chromatin with high-throughput sequencing (ATAC-seq) have shown that the epigenetic landscape of exhausted CD8^+^ T cells is distinct from that of effector and memory CD8^+^ T cells [89, 90]. Remarkable differences in the accessible chromatin regions were observed between exhausted CD8^+^ T cells and effector/memory CD8^+^ T cells [89, 90]. For example, several open chromatin regions in the *Ifng* locus are present in effector and memory CD8^+^ T cells but not in exhausted CD8^+^ T cells [90]. In contrast, open chromatin regions related to IRs, such as PD-1, are specific to exhausted CD8^+^ T cells [89, 90].

### Metabolic reprogramming

The activation and clonal expansion of CD8^+^ T cells are accompanied by alterations in cellular metabolism. During acute infection, a transition from mitochondrial oxidative phosphorylation to glycolysis is required for differentiation into effector CD8^+^ T cells [91–93]. Memory precursor T cells alter their cellular metabolism to oxidative phosphorylation and fatty acid oxidation [94]. In transcriptomic analysis, substantial alterations have been observed in genes involved in metabolism and bioenergetic pathways in exhausted CD8^+^ T cells, suggesting that CD8^+^ T-cell exhaustion is accompanied by metabolic alterations [79]. Exhausted CD8^+^ T cells are known to undergo metabolic reprogramming, including decreased glycolysis and dysregulated mitochondrial energetics [95]. Moreover, PD-1 signaling suppresses glycolysis and promotes fatty acid oxidation in CD8^+^ T cells by inhibiting PI3K/Akt and MEK/ERK signaling [96]. Furthermore, PD-1 blockade restores glycolysis in exhausted CD8^+^ T cells [97].

## Uncoupling T-cell exhaustion from activation

Considering that CD8^+^ T-cell exhaustion results from persistent stimulation of T cells, it is challenging to distinguish T-cell exhaustion from activation. The surface markers and transcriptional signatures of exhausted CD8^+^ T cells closely overlap with those of activated CD8^+^ T cells [88, 98–100]. In addition, most characteristics of CD8^+^ T-cell exhaustion are individually insufficient to identify exhausted CD8^+^ T cells. In particular, because the majority of IRs are also transiently expressed in effector CD8^+^ T cells during activation, IR expression is not a unique feature of exhausted CD8^+^ T cells [44, 101]. A previous study also showed no impairment of cytokine production in CD8^+^ T cells expressing a diverse array of IRs, indicating that IR expression may not be directly linked to dysfunction [102]. In transcriptomic analyses of CD8^+^ tumor-infiltrating lymphocytes from tumor-bearing mice, many IRs are present in the activation/dysfunction gene module but not in the dysfunctional gene module [103]. Furthermore, genes related to the cell cycle pathway, migration, cytotoxic molecules, and costimulatory receptors are commonly upregulated in both exhausted and activated CD8^+^ T cells [79].

Therefore, simultaneous consideration of diverse features, including dysfunction, sustained IR expression, transcriptional and epigenetic alterations, and metabolic derangement, is needed to identify *bona fide* exhausted CD8^+^ T cells and uncouple them from activated CD8^+^ T cells.

## An overview of CD8^+^ T-cell responses against SARS-CoV-2 in patients with COVID-19

Since the outbreak of COVID-19, we have gained much information about CD8^+^ T-cell responses to SARS-CoV-2. Early studies reported that SARS-CoV-2-specific CD8^+^ T-cell responses are successfully elicited by SARS-CoV-2 infection [12, 13, 17]. SARS-CoV-2-specific CD8^+^ T-cell responses have been identified in ~70% of convalescent individuals after recovery from COVID-19 [12]. These responses are specific to a wide range of SARS-CoV-2 antigens, including spike, nucleocapsid, and membranous proteins, as well as other nonstructural proteins [12, 13, 17].

A series of studies suggest a critical role of CD8^+^ T cells in protecting against the development of severe COVID-19. SARS-CoV-2-specific CD8^+^ T-cell responses correlate with low disease severity during the acute phase [104]. Memory T-cell responses have been detected in COVID-19 convalescent individuals even in the absence of SARS-CoV-2-specific antibodies [105]. In addition, CD8^+^ T cells from the bronchoalveolar lavage fluid of patients with severe/critical COVID-19 exhibit a lack of dominant clones compared to those from the bronchoalveolar lavage fluid of patients with mild disease [106].

Recently, studies using animal models revealed the importance of CD8^+^ T cells in controlling SARS-CoV-2 infection. Limited viral clearance in the respiratory tract was observed in CD8^+^-depleted convalescent rhesus macaques upon SARS-CoV-2 rechallenge, implying that memory CD8^+^ T cells are required for the clearance of SARS-CoV-2 [18]. Furthermore, T-cell vaccination that does not elicit neutralizing antibodies partially protects SARS-CoV-2-infected mice from severe disease [19].

## The CD8^+^ T-cell population in patients with COVID-19

### The upregulation of activation markers and inhibitory receptors

There is a growing body of evidence that circulating CD8^+^ T cells from patients with severe COVID-19 exhibit an activated phenotype characterized by increased expression of CD38, HLA-DR, and Ki-67 [16, 20–22, 107]. In addition, a recent study analyzing airway immune cells revealed that CD8^+^ T cells from the airways of patients with COVID-19 were predominantly tissue-resident memory T cells and that these cells have an elevated proportion of activated cells [108].

An exhausted CD8^+^ T-cell phenotype with an upregulation of IRs, such as PD-1, TIM-3, LAG-3, CTLA-4, NKG2A, and CD39, has been described in patients with COVID-19, particularly in those with severe disease [20, 25–29]. In addition, an scRNA-seq analysis of peripheral blood mononuclear cells (PBMCs) showed that the exhaustion score in the CD8^+^ effector cluster was significantly higher in patients with severe COVID-19 than in healthy donors and patients with moderate disease [109]. Moreover, increased PD-L1 expression has been reported in basophils and eosinophils from patients with severe COVID-19 [110].

In contrast, a number of studies have reported no evidence of CD8^+^ T-cell exhaustion in patients with COVID-19, even in those with severe cases. An early study performing scRNA-seq analysis of PBMCs found that the T-cell exhaustion module score was not significantly changed in CD8^+^ T cells from patients with COVID-19, even in patients with severe cases with acute respiratory distress syndrome, compared to healthy donors [31]. In addition, a recent study using single-cell cellular indexing of transcriptomes and epitopes by sequencing (CITE-seq) and TCR sequencing described that a cluster of exhausted CD8^+^ T cells was not associated with COVID-19 [32]. In that study, the exhaustion of clonally expanded CD8^+^ T cells, as evaluated by IR expression, was not associated with disease severity [32].

Discrepancies in the results may be derived from several factors. First, there were differences in the criteria for disease severity among studies. Second, the exhaustion gene sets used in the analysis or the detailed method of analysis for the transcriptomic data were different. Third, the demographics of the study cohorts need to be considered.

### The functions of CD8^+^ T cells in patients with COVID-19

Several studies have reported that CD8^+^ T cells from patients with COVID-19 exhibit a decreased cytokine-producing capacity upon stimulation with PMA/ionomycin [23, 27]. In contrast, another study reported that CD8^+^ T cells from patients with COVID-19 exert higher effector functions, including the production of IL-2 and IL-17A and the expression of the degranulation marker CD107a, upon anti-CD3/CD28 stimulation compared to cells from healthy donors [25]. However, these studies examined the functions of the CD8^+^ T-cell population following ex vivo stimulation with pan-T cell stimulants, not SARS-CoV-2 antigens; thus, they lack information on the antigen specificity of CD8^+^ T cells.

## SARS-CoV-2-specific CD8^+^ T cells in patients with COVID-19

### The phenotype of SARS-CoV-2-specific CD8^+^ T cells

**Fig. 2:**
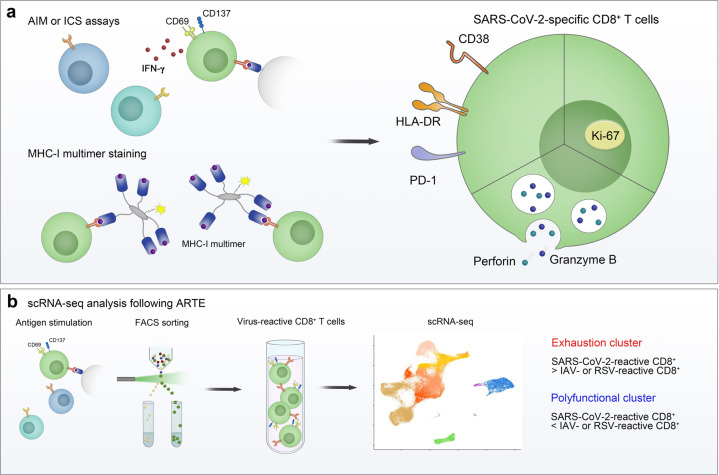
Phenotype of SARS-CoV-2-specific CD8^+^ T cells. The phenotype of SARS-CoV-2-specific CD8^+^ T cells was examined using **a** ex vivo stimulation-based functional assays, MHC-I multimer staining, and **b** single-cell RNA sequencing (scRNA-seq) following antigen-reactive T-cell enrichment (ARTE). In the acute phase, SARS-CoV-2-specific CD8^+^ T cells express activation markers (CD38 and HLA-DR), PD-1, Ki-67, and cytotoxic proteins (perforin and granzyme B). In scRNA-seq analysis of virus-reactive CD8^+^ T cells, the proportion of the “exhaustion” cluster, characterized by increased expression of exhaustion-associated genes, was higher in SARS-CoV-2-reactive CD8^+^ T cells than in influenza A virus (IAV)- or respiratory syncytial virus (RSV)-reactive CD8^+^ T cells. In addition, the proportion of the “polyfunctional” cluster expressing high levels of genes encoding cytokines was lower in SARS-CoV-2-reactive CD8^+^ T cells than in IAV- or RSV-reactive CD8^+^ T cells. AIM activation-induced marker, ICS intracellular cytokine staining

Considering that only a proportion of the CD8^+^ T-cell population is specific to the infecting virus, it is important to examine the phenotype and functions of viral-antigen-specific CD8^+^ T cells, not the total CD8^+^ T cell population, during viral infection. SARS-CoV-2-specific CD8^+^ T cells from COVID-19 patients have been investigated by many researchers (Fig. 2). Early studies examined SARS-CoV-2-specific CD8^+^ T-cell responses using ex vivo stimulation-based functional assays, such as intracellular cytokine staining and activation-induced marker assays [12, 13, 15, 17]. In addition, scRNA-seq analysis following antigen-reactive T-cell enrichment (ARTE) allowed us to investigate SARS-CoV-2-reactive CD8^+^ T cells at the transcriptome level [111, 112]. However, all of these assays have inherent limitations in that ex vivo stimulation may change the phenotype of CD8^+^ T cells. Moreover, stimulation-based functional assays detect functioning T cells, not virus-specific nonfunctioning cells. In contrast, MHC multimer techniques, which directly detect antigen-specific T cells, do not have these caveats [113]. Several studies using MHC-I multimers have examined the phenotypes of SARS-CoV-2-specific CD8^+^ T cells [16, 17, 33, 114, 115].

In the acute phase of COVID-19, SARS-CoV-2-specific MHC-I multimer^+^CD8^+^ T cells express activation markers (CD38 and HLA-DR), Ki-67, IRs (PD-1, TIM-3, and LAG-3), and cytotoxic proteins (perforin and granzyme B), indicating that these cells are activated and proliferate with a high cytotoxic capacity [16]. This finding is in line with the result that SARS-CoV-2-reactive CD8^+^ T cells detected by stimulation-based assays express CD38, HLA-DR, Ki-67, and PD-1 [16]. Similar results were observed in our analysis with MHC-I multimer staining. In a longitudinal analysis, we found that the expression of PD-1 and CD38 in MHC-I multimer^+^ cells decreases during the course of COVID-19 [33]. We also observed an inverse correlation between the expression of PD-1 and CD38 in MHC-I multimer^+^ cells and the number of days since symptom onset. These kinetics suggest that PD-1 expression in SARS-CoV-2-specific CD8^+^ T cells is transient, not persistent, in patients with COVID-19. Thus far, relatively few studies have examined the expression of IRs other than PD-1 in SARS-CoV-2-specific CD8^+^ T cells. In the acute phase of severe COVID-19, a considerable proportion of SARS-CoV-2-specific CD8^+^ T cells express TIM-3, LAG-3, TIGIT, and CTLA-4 [16]. The expression of TIM-3 and TIGIT in SARS-CoV-2-specific CD8^+^ T cells tended to be lower among patients who recovered from mild COVID-19 than among patients with acute severe COVID-19 [16].

A recent scRNA-seq analysis of virus-reactive CD8^+^ T cells obtained using ARTE demonstrated that the proportion of the “exhaustion” CD8^+^ T-cell cluster characterized by increased expression of exhaustion-associated genes, including *HAVCR2* (TIM-3) and *LAG3*, was higher in SARS-CoV-2-reactive CD8^+^ T cells from COVID-19 patients than in influenza A virus (IAV)- and respiratory syncytial virus (RSV)-reactive CD8^+^ T cells from healthy donors [111]. Intriguingly, the exhaustion cluster showed significant enrichment of cytotoxicity-related genes, such as *GZMB*, *GZMA*, *GZMH*, *PRF1*, and *TBX21*, indicating that this cluster is not associated with dysfunction. On the other hand, the proportion of the cluster expressing high levels of genes encoding cytokines, including *IFNG*, *TNF*, *CCL3*, *CCL4*, *XCL1*, and *XCL2*, was lower in SARS-CoV-2-reactive CD8^+^ T cells from COVID-19 patients than in IAV- and RSV-reactive CD8^+^ T cells from healthy donors [111], suggesting that SARS-CoV-2-reactive CD8^+^ T cells have a reduced capacity to secrete effector cytokines.

### The functions of PD-1-expressing SARS-CoV-2-specific CD8^+^ T cells

**Fig. 3:**
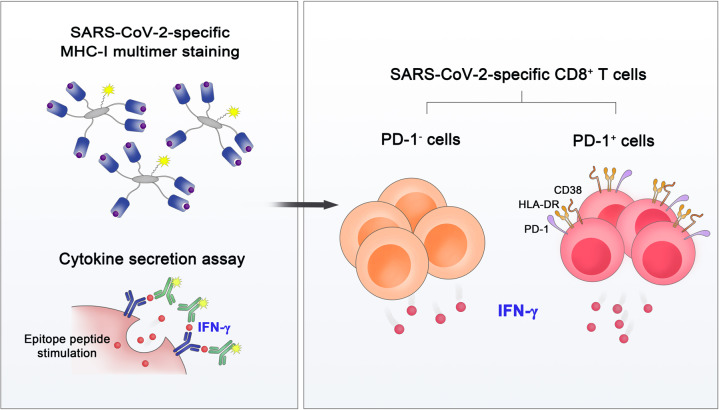
Functional analysis of SARS-CoV-2-specific MHC-I multimer^+^CD8^+^ T cells. MHC-I multimer staining in combination with cytokine secretion assays revealed that both PD-1^+^ and PD-1^–^ cells among SARS-CoV-2-specific CD8^+^ T cells are functional in terms of IFN-γ production

To investigate the effector functions of SARS-CoV-2-specific CD8^+^ T cells, our group performed MHC-I multimer staining, followed by proliferation assays and cytokine secretion assays (Fig. 3) [33]. SARS-CoV-2-specific MHC-I multimer^+^ T cells from individuals who recovered from COVID-19 showed robust proliferation upon ex vivo antigen challenge. In addition, despite the lower frequency of IFN-γ-producing cells in SARS-CoV-2-specific CD8^+^ T cells than IAV-specific CD8^+^ T cells, IFN-γ was produced by SARS-CoV-2-specific CD8^+^ T cells regardless of their PD-1 expression. The same results were observed when we analyzed SARS-CoV-2-specific MHC-I multimer^+^ cells from acute COVID-19 patients. These findings indicate that PD-1^+^ cells among SARS-CoV-2-specific MHC-I multimer^+^ cells are not exhausted but functionally active in the acute and early convalescent phases of COVID-19 and that PD-1 needs to be considered an activation marker rather than an exhaustion marker in patients with COVID-19. In addition, there was no significant difference between patients with severe and nonsevere COVID-19 in regard to IFN-γ production by SARS-CoV-2-specific MHC-I multimer^+^CD8^+^ T cells. However, our study relied on MHC-I multimers specific to HLA-A*02-restricted epitopes from the spike protein. CD8^+^ T cells specific to other SARS-CoV-2 epitopes restricted by other HLA-I allotypes may differ in phenotype and function. In addition, given that the impairment of IFN-γ production occurs in the later stage of T-cell exhaustion [55], the production capacities of other cytokines, such as IL-2 and TNF, and cytotoxicity need to be examined further in SARS-CoV-2-specific MHC-I multimer^+^CD8^+^ T cells.

Thus far, the phenotype and functions of SARS-CoV-2-specific CD8^+^ T cells have been analyzed primarily in peripheral blood [16, 17, 33, 114, 115]. However, previous studies in animal models of respiratory viral infections have shown that tissue-resident memory T cells in the respiratory tract critically contribute to protection from viral infection [116, 117]. In patients with COVID-19, the expression of tissue-residency markers (CD69 and CD103) and activation markers (PD-1 and HLA-DR) is higher in airway CD8^+^ T cells than in their peripheral blood counterparts [108], indicating that tissue-resident CD8^+^ T cells with an activated phenotype are enriched in the airways. Therefore, additional studies are needed to investigate whether SARS-CoV-2-specific CD8^+^ T cells in the respiratory tract are exhausted or functional in patients with COVID-19.

Moreover, comprehensive investigations on the transcriptional and epigenetic dynamics of SARS-CoV-2-specific CD8^+^ T cells would provide new insights into the differentiation trajectories of CD8^+^ T cells and clarify whether CD8^+^ T cells are truly exhausted during the course of COVID-19.

### The development of SARS-CoV-2-specific T-cell memory

Accumulating evidence suggests that SARS-CoV-2-specific T-cell responses are maintained in convalescent individuals up to 10 months post infection, indicating that SARS-CoV-2-specific T-cell memory develops successfully and is long lasting [118–124]. As CD8^+^ T cells that fail to become functional memory T cells differentiate into exhausted T cells, these findings suggest that CD8^+^ T-cell exhaustion may be limited in the majority of patients with COVID-19.

Among subsets of memory T cells, stem cell-like memory T (T_SCM_) cells are characterized by a high self-renewal capacity and a multipotent ability to generate diverse memory subsets [125, 126]. Stem-like CD8^+^ memory T-cell progenitors have been described as being composed of two distinct subsets based on PD-1 and TIGIT expression [127]. Our group recently showed that the majority of SARS-CoV-2-specific T_SCM_ cells from convalescent COVID-19 patients are PD-1^–^TIGIT^–^ cells, suggesting that these cells are not exhausted-like progenitors [124]. These findings also support SARS-CoV-2-specific CD8^+^ T cells being rarely exhausted in patients with COVID-19. Limited exhaustion of SARS-CoV-2-specific CD8^+^ T cells and successful development of T_SCM_ cells lead to host protection upon re-exposure to SARS-CoV-2 among COVID-19 convalescent individuals.

### CD8^+^ T-cell exhaustion and vaccine-induced memory T-cell responses

Currently available vaccines using diverse platforms have been shown to elicit protective T-cell immunity [4, 7, 128–130]. Currently, COVID-19 vaccines are administered not only to unexposed individuals but also to COVID-19 convalescent individuals. Given that exhausted CD8^+^ T cells lose their potential to differentiate into memory T cells, the potential CD8^+^ T-cell exhaustion in individuals who have had COVID-19 can impede vaccine-induced development of T-cell memory. However, because CD8^+^ T-cell exhaustion is not evident in patients with COVID-19, it is assumed that COVID-19-experienced individuals successfully develop functional CD8^+^ T-cell memory following vaccination. Recent studies have reported that a single dose of mRNA vaccine robustly induces spike-specific T-cell responses in COVID-19 convalescent individuals [131].

## The exhausted-like phenotypes of CD8^+^ T cells in respiratory viral infections

An exhausted-like phenotype of CD8^+^ T cells has been reported in several studies of respiratory viral infections using mouse models. PD-1 upregulation on virus-specific CD8^+^ T cells and an impairment of their effector functions have been observed during infection with respiratory viruses, such as human metapneumovirus or influenza virus [132–134]. Similar to T-cell exhaustion during chronic viral infections, the PD-1 pathway primarily mediates functional impairment of CD8^+^ T cells in acute respiratory virus infection [132, 134]. However, this functional alteration occurs more rapidly than T-cell exhaustion [132]. Furthermore, whether the differentiation state and transcriptional profiles of functionally impaired CD8^+^ T cells in respiratory viral infections are similar to those of exhausted T cells is not clear. Before the COVID-19 pandemic, little was known about the functional impairment or exhaustion of CD8^+^ T cells during respiratory viral infections in humans. Further investigations with functional, transcriptomic, epigenetic, and metabolic profiling are needed to clarify T-cell exhaustion in acute respiratory viral infections.

## Concluding remarks and perspectives

Since the emergence of COVID-19, global efforts have rapidly increased our knowledge of the immune responses to SARS-CoV-2, including CD8^+^ T-cell responses. However, information regarding the role of SARS-CoV-2-specific CD8^+^ T cells in protective immunity is still limited. In addition, the differentiation dynamics of CD8^+^ T cells during the course of COVID-19, particularly whether SARS-CoV-2-specific CD8^+^ T cells become exhausted, remain enigmatic. Further comprehensive studies on the functional, transcriptional, epigenetic, and metabolic landscapes of SARS-CoV-2-specific CD8^+^ T cells would help answer this question. Moreover, considering that virus-specific effector T cells are recruited to the site of inflammation, SARS-CoV-2-specific CD8^+^ T cells in the respiratory tract should be investigated. Deeper investigation of CD8^+^ T cells will help not only control the ongoing COVID-19 pandemic but also prepare for any upcoming pandemics.
